# A Randomized, Double-Blind, Placebo-Controlled, Two-Way Crossover Clinical Trial of ORADUR-Methylphenidate for Treating Children and Adolescents with Attention-Deficit/Hyperactivity Disorder

**DOI:** 10.1089/cap.2020.0104

**Published:** 2021-04-16

**Authors:** Yu-Shu Huang, Chin-Bin Yeh, Chin-Hung Chen, Chi-Yung Shang, Susan Shur-Fen Gau

**Affiliations:** ^1^ Department of Child Psychiatry, Chang Gung Memorial Hospital and University, Taoyuan, Taiwan.; ^2^ Department of Psychiatry, Tri-Service General Hospital and University, Taipei, Taiwan.; ^3^ Department of Psychiatry, Chang Gung Memorial Hospital and University, Chiayi, Taiwan.; ^4^ Department of Psychiatry, National Taiwan University Hospital and College of Medicine, National Taiwan University, Taipei, Taiwan.

**Keywords:** attention-deficit/hyperactivity disorder, children, ORADUR-methylphenidate, efficacy, safety

## Abstract

***Objective:*** Methylphenidate (MPH) is efficacious in reducing symptoms of attention-deficit/hyperactivity disorder (ADHD), but there are no data about the efficacy and safety of its new formulation (ORADUR^®^-MPH extended release, ORADUR-MPH) in patients with ADHD, which is the study objective.

***Method:*** This was a Phase III, multicenter, randomized, double-blind, placebo-controlled, two-way crossover clinical trial. One hundred children and adolescents with a clinical diagnosis of ADHD (72.7% male) received at least one dose of ORADUR-MPH or a placebo during the 2-week treatment period of each phase. The primary efficacy measure was the Swanson, Nolan, and Pelham-IV-teacher (SNAP-IV-T) form. Secondary efficacy measures included the SNAP-IV-parent form, the Clinical Global Impression: ADHD-Severity score, the Conner's Teacher's Rating Scale score, and the investigator's rating for 18 Diagnostic and Statistical Manual of Mental Disorders, 5th edition ADHD symptoms. In addition, data related to vital signs, body weight, physical examination, laboratory testing, and adverse events (AEs) were also collected. All data were analyzed on an intent-to-treat basis.

***Results:*** Without adjusting for differences in demographics and baseline measures, both treatment groups showed significant reductions in ADHD and oppositional defiant disorder symptoms after a 2-week treatment with greater effect sizes (Cohen's *d*) in the ORADUR-MPH group (Cohen's *d* ranging from −0.41 to −1.64; placebo, Cohen's *d* ranging from −0.26 to −1.18), except for oppositional symptoms, regardless of the informants. For the primary efficacy measure, ORADUR-MPH was significantly superior to the placebo, as evidenced by lower values for and greater reductions in the SNAP-IV-T scores at the endpoint (Cohen's *d* = −0.16, *p* = 0.005) and from baseline to the endpoint (Cohen's *d* = −0.19, *p* = 0.006), respectively. There were no serious AEs during the clinical study period. The most frequently observed AE was decreased appetite (49.1%). Most physical and laboratory test variables remained within the normal range.

***Conclusions:*** Once-daily ORADUR-MPH is an effective, well-tolerable, and safe treatment for children and adolescents with ADHD. ClinicalTrials.gov number, NCT02450890.

## Introduction

Attention-deficit/hyperactivity disorder (ADHD), which is characterized by developmentally inappropriate inattention, hyperactivity, and impulsivity, is a common neuropsychiatric disorder that affects 2%–7% of individuals all over the world (Sayal et al. 2018); the frequency of this disorder is higher in the United States and Taiwan, being 8.5%–9.5% (Zablotsky et al. 2019) and 7%–8% (Gau et al. 2005; Chen et al. 2019), respectively. ADHD has a strong neurobiological basis and is supported by extensive research involving neuropsychology, neuroimaging, and genetics (Faraone et al. 2015; Demontis et al. 2019; Hearne et al. 2019; Shang et al. 2019). The core symptoms of ADHD last until adulthood (Reimherr et al. 2015) and are associated with a high frequency of psychiatric comorbid conditions during both adolescence (Gau et al. 2010) and adulthood (Lin et al. 2016); these are associated with long-term functional impairment and reduced life quality (Lin et al. 2015). Due to its high prevalence, the pervasiveness of the impairment, and its tremendous impact on the individual, their family, and society in general, treatment of ADHD, which can include pharmacotherapy (Nageye and Cortese 2019) and psychosocial interventions, is crucial (Faraone et al. 2015).

Psychostimulants and atomoxetine are two medications that have been approved to treat ADHD (Cortese et al. 2018; Nageye and Cortese 2019), and these two treatments have different mechanisms (Shang et al. 2016). Methylphenidate (MPH), a dopamine and noradrenaline reuptake inhibitor (Faraone 2018), is the recommended first-line pharmacological treatment for ADHD in many countries (Conners 2002; Agster et al. 2011; Cortese et al. 2017), including Taiwan (Gau et al. 2008), with treatment response rates being between 70% and 90% (Goldman et al. 1998). MPH is one of the most commonly used psychostimulants and therefore has been the most widely studied regarding its efficacy when treating ADHD worldwide (Cortese et al. 2017, 2018). Converging evidence supports the conclusion that MPH efficaciously reduces ADHD symptoms (Gau et al. 2006a) and other related emotional and behavioral symptoms (Shih et al. 2019) in children and adolescents with ADHD, while at the same time improving academic achievement (Kortekaas-Rijlaarsdam et al. 2019), social functioning (Shang et al. 2019), neuropsychological performance (Chou et al. 2015), and brain functioning (Shang et al. 2016).

There are several available formulations of the two stimulant classes, including immediate-release, short-duration, and extended-release medications (Hazell 2007; Childress 2017; Cortese et al. 2018; Faraone 2018). Both immediate-release MPH (IR-MPH) and osmotic-release oral system MPH (OROS-MPH) are effective when treating children with ADHD (Pelham et al. 2001; Huang et al. 2011). However, given that IR-MPH may bring about decreased adherence to treatment, some studies have suggested that OROS-MPH is superior to IR-MPH when treating ADHD due to the dosing schedule (Gau et al. 2006b, 2008; Steele et al. 2006). Despite a number of long-acting formulations being available on the market, such as OROS-MPH, several drug companies have been developing novel long-acting formulations that they hope will improve adherence, reduce abuse potential, decrease stigma, and decrease the likelihood of adverse effects related to a dosage peak (Cortese et al. 2017). Among these newly developed long-acting formulations, ORADUR technology, a once-daily tamper-resistant formulation that allows MPH sustained release, was developed to fulfill the unmet needs of patients treated using current formulations (Cortese et al. 2017, 2018).

ORADUR^®^-MPH involves a once-a-day dose and includes a high-viscosity base component. ORADUR technology focused on addressing two interrelated challenges; these are extending the therapeutic duration of conventional short-acting drugs and protecting the required high drug loads from tampering and improper extraction. According to a previous study (Swanson and Volkow 2002), the maximum blockade of dopamine transporters (DATs) by MPH is achieved at serum levels between 8 and 10 ng/mL. A pharmacokinetic study of ORADUR-MPH in healthy adults revealed that, when taken orally, either 44 mg of ORADUR-MPH or 36 mg of OROS-MPH is able to reach an effective serum concentration, namely 10.351 and 8.214 ng/mL, respectively, using multiple dose (QPS 2013). The data suggest that ORADUR-MPH might be more effective at inhibiting DATs than OROS-MPH. With the shorter and the higher, the absorption rate of ORADUR-MPH is faster compared with OROS-MPH.

At present, ORADUR-MPH has not been approved by the Food and Drug Administration for marketing in the United States for any indication. Nevertheless, we hypothesize that, like other long-acting formulation of MPH, ORADUR-MPH should be able to provide therapeutic benefit by improving the core symptoms of ADHD and related clinical symptoms. Hence, we have conducted a multicenter, randomized, double-blind, placebo-controlled, two-way crossover clinical trial to evaluate the short-term efficacy and safety of ORADUR-MPH when treating children with ADHD.

## Methods

### Participants

Children and adolescents with a clinical diagnosis of ADHD, who were between 6 and 18 years of age, were recruited from the Department of Psychiatry, National Taiwan University, Taipei, Taiwan, the Department of Psychiatry, Tri-Service General Hospital and University, Taipei, Taiwan, and the Department of Child Psychiatry, Chang Gung Memorial Hospital, Linkou, Taiwan. Each clinical diagnosis was made by a senior child psychiatrist according to the Diagnostic and Statistical Manual of Mental Disorders, 5th edition (DSM-5) diagnostic criteria for ADHD (American Psychiatric Association 2013), and this diagnosis was further confirmed by psychiatric interview using the Chinese version of the Kiddie Schedule for Affective Disorders and Schizophrenia for School-Age Children-Epidemiological (K-SADS-E) Version, which is used for the diagnosis of ADHD and other psychiatric disorders (Gau et al. 2005; Chen et al. 2017). The Chinese K-SADS-E has been proved to be a reliable and valid instrument when assessing childhood psychiatric disorders and has been used extensively in Taiwan in various studies that have targeted childhood mental disorders (Shang et al. 2016, 2017, 2019; Chiang et al. 2019; Hearne et al. 2019; Lin and Gau 2019; Lin et al. 2020).

The inclusion criteria were as follows: (i) children and adolescents, between 6 and 18 years of age, who had been clinically diagnosed with ADHD according to the DSM-5 criteria within that last year; (ii) children and adolescents who were able to swallow the study-specific capsule (18 mm) without difficulty; and (iii) participants and their parents/guardians who were able to provide their written informed consent. Participants were excluded if they had received ADHD treatment for over 1 year or had received ADHD treatment within 30 days before the study treatment initiation; if they were known to be allergic to any of the ORADUR-MPH ingredients; if their intelligence quotient (IQ) was <80; if they had taken any psychotropic drug concomitantly within the 14 days before the study treatment initiation; if they had glaucoma (narrow-angle glaucoma), an on-going seizure disorder, any systemic disease, any disorder involving tics, or any other psychotic disorders; if they or their caregiver(s) had exhibited drug or alcohol abuse/dependence within the prior 6 months; or if there seemed to be any condition associated with the individual that potentially hampered adherence to the study protocol and follow-up schedule.

### Procedures

This study was approved by each site's Research Ethics Committee or Institutional Review Board (approval number: 201412007MSB, 2-103-1-009, 104-2372A). Before carrying out any study of the procedures or dispensing of study drugs, all the participants and their parents provided written informed consent; this was done after the procedures and duration of the study had been explained; volunteer participation was fully ensured.

**FIG. 1.**
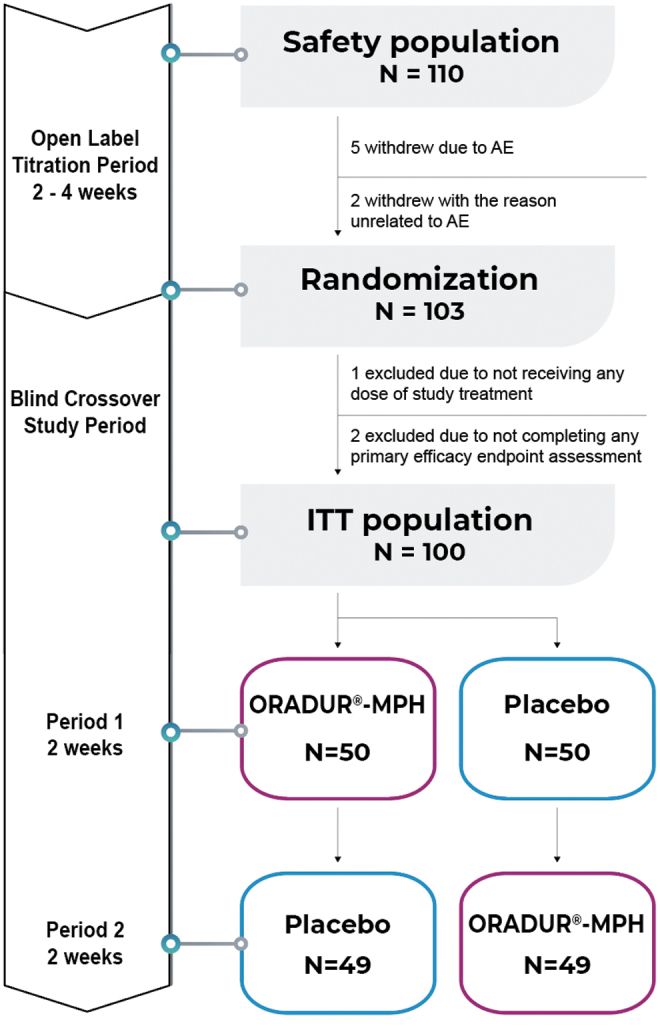
Flowchart of the sampling process, the randomization procedure, and the crossover design.

The study consisted of four phases, and these were as follows: (i) the screening period, which lasted about 14 days, (ii) the open-label titration period, which lasted 2–4 weeks, (iii) the double-blinded and placebo-controlled two-way crossover treatment phase, which lasted 4 weeks, and (iv) the follow-up phase, which lasted 2 weeks (Fig. 1). The study drug was an ORADUR-MPH oral capsule that had three dosage formats (22, 33, and 44 mg). Both ORADUR-MPH and the placebo were orally administered once daily in the morning in the 20 minutes after breakfast. The initial dose for all enrolled participants was 22 mg for 1 week. The dosage was then titrated according to the clinical response and presence of adverse effects. After the optimal dosage had been determined, each participant received additional 1-week treatment at this optimal dosage before randomization. Participants who were unable to tolerate 22 mg ORADUR-MPH were withdrawn.

Based on a computer-generated random sequence, the study participants were randomly assigned into either the ORADUR-MPH or the placebo parts of the trial at a 1:1 ratio that involved a blinded crossover treatment phase for 4 weeks (2 weeks for each, Period 1 and Period 2) using two different sequences, namely ORADUR-MPH-placebo and placebo-ORADUR-MPH. This crossover treatment phase was carried out without a washout period between Period 1 and Period 2.

Bodyweight, body height, blood pressure, heart rate, the score for the ADHD supplement of the K-SADS-E, and Clinical Global Impression-ADHD severity (CGI-ADHD-S) were assessed by the investigators, teacher reports, and parent reports using the Chinese version of the Swanson, Nolan, and Pelman-IV (SNAP-IV-teacher [SNAP-IV-T] and SNAP-IV-parent [SNAP-IV-P], respectively). Furthermore, teacher reports using the Conner's Teacher's Rating Scale-Revised: Short (CTRS-R: S) Form for ADHD symptoms were gathered at each visit, starting at baseline (before randomization for crossover Period 1) through to the endpoints of Period 1 (week 2) and of Period 2 (week 4). The side effects of medications were investigated at the endpoints of both Period 1 and Period 2 (Fig. 1).

#### Primary efficacy measure

##### Swanson, Nolan, and Pelman-IV

The SNAP-IV, a 26-item scale, consists of inattention (item 1–9), hyperactivity/impulsivity (item 10–18), and oppositionality (item 19–26) items that correspond to the core symptoms of Diagnostic and Statistical Manual of Mental Disorders, 4th edition (DSM-IV; American Psychiatric Association 1994) ADHD and oppositional defiant disorder (ODD) (Swanson et al. 2001). The 26 items of the SNAP-IV are rated on a 4-point Likert scale: “0 for not at all,” “1 for just a little,” “2 for quite a bit,” and “3 for very much.” The psychometric properties of the Chinese SNAP-IV-P (Gau et al. 2008) and SNAP-IV-T (Gau et al. 2009) have been established, and they show excellent validity and reliability; they have been widely used in a range of clinical trials assessing the efficacy of medications when treating ADHD (Gau et al. 2008; Shang et al. 2015), in various epidemiological studies (Chen et al. 2015; Tsai et al. 2017), and in clinical research (Chiang et al. 2015, 2016, 2019; Shang et al. 2015; Chiang and Gau 2016). The Chinese SNAP-IV-T and SNAP-IV-P are the primary and secondary efficacy measures of this study, respectively.

#### Secondary efficacy measures

The secondary outcome measures included the CGI-ADHD-S and the ADHD symptoms (K-SADS-E) that were assessed by the investigators, and the Chinese SNAP-IV-P and the Chinese CTRS-R: S.

##### Clinical Global Impression-ADHD severity

The CGI-ADHD-S, which used investigator rating, is a single-item rating of a clinician's assessment of the global severity of ADHD symptoms and is related to the clinician's total experience with other ADHD patients. The severity is rated on a 7-point scale with the extremes of 1 and 7 representing the ratings normal, not at all ill, and most extremely ill. The Chinese CGI-ADHD-S has been used widely in various treatment studies related to ADHD in Taiwan (Gau et al. 2007; Martenyi et al. 2009; Gau and Shang 2010; Shang et al. 2015).

##### Kiddie Schedule for Affective Disorders and Schizophrenia for School-Age Children-Epidemiological

The K-SADS-E is a semistructured interview-based scale for the systematic assessment of mental disorders affecting children and adolescents. The development of the Chinese K-SADS-E was carried out by the Child Psychiatry Research Group in Taiwan (Gau and Soong 1999). This work included a two-stage translation and modification of several items into psycholinguistic equivalents relevant to Taiwanese culture, and this was followed by a further modification to match the DSM-IV (Gau et al. 2005) and DSM-5 (Chen et al. 2017) diagnostic criteria. Previous studies have shown that the Chinese K-SADS-E is a reliable and valid instrument for assessing child and adolescent psychiatric disorders (Gau et al. 2005). It has been extensively used in a variety of clinical studies (Chou et al. 2015), epidemiological studies (Chen et al. 2019), neuropsychological studies (Hwang-Gu and Gau 2015), and neuroimaging studies (Chiang et al. 2019; Shang et al. 2019, 2020a, 2020b). K-SADS-E interview training has been described in detail elsewhere (Gau et al. 2010; Chen et al. 2017). The 18 symptom items that form the ADHD supplement of the K-SADS-E were assessed by the investigators at baseline, at the endpoint of Period 1, and the endpoint of Period 2.

### Conner's Teacher's Rating Scale-Revised: Short

The CTRS-R: S, a 28-item teacher-reported rating scale, consists of three subscales: Oppositional (5 items), Cognitive Problems/Inattention (5 items), and Hyperactivity/Impulsivity (7 items); and the ADHD-index (12 items) (Conners 1997; Conners et al. 1998). Each item is rated on a 4-point Likert scale with 0 for not true at all (never or seldom), 1 for just a little true (occasionally), 2 for pretty much true (often or quite a bit), and 3 for very much true (very often or very frequent) (Conners 1997). The Chinese version of the CTRS-R: S has been demonstrated to be a reliable and valid instrument for measuring inattention and hyperactivity in an ethnic Chinese population (Gau et al. 2006c). The CTRS-R: S has satisfactory factor structures, as well as adequate test–retest reliability, internal consistency, and convergent validity, and can clearly distinguish children and adolescents with ADHD from those without (Gau et al. 2006c). The Chinese version of the CTRS-R: S had been used to assess ADHD-related symptoms in clinical research in Taiwan (Gau 2006; Gau et al. 2006a, 2006b, 2007; Chiang et al. 2010; Tseng et al. 2011; Kawabata et al. 2012).

#### Safety measures

Safety and tolerability were assessed at each visit through open-ended questions during an initial clinical interview by the investigators, and this was followed by a structured interview by the investigators based on a standard questionnaire that includes all potential adverse effects. The targeted treatment-emergent adverse events (TEAEs) included decreased appetite, vomiting, insomnia, somnolence, dizziness, stomach aches, headaches, palpitation, and a dry mouth. Potentially serious adverse events (AEs) and vital signs were assessed at each visit through blood pressure measure, heart rate measurement, body weight assessment, and physical examination findings. A 12-lead electrocardiogram and various laboratory tests (included hematology, blood biochemistry, urinalysis, and urine pregnancy test) were also assessed at baseline and endpoint.

#### Adherence

We employed both subjective and objective assessments to determine whether the participants were adhering to the medication regime. Parents and participants provided their retrospective feedback on adherence as the subjective assessment. The objective evaluation of daily adherence and the frequency of missed doses were based on a pill count by a research assistant and a standard interview conducted by the investigators. If the information collected through the two methods was not consistent with each other, the investigators would interview the parents and participants again and discuss the difference between parent reports, self-reports, and pill count, and then determine the number of days that the medication had been taken.

#### Data analysis

Sample size estimation was calculated based on previous clinical studies (Steele et al. 2006; Szobot et al. 2008; Chou et al. 2012). By assuming that the mean difference between MPH and the placebo in terms of the SNAP-IV total score at 2 weeks was to be −6.0, the individual standard deviations (SDs) would have been 10.0. Based on such information, the total sample size was estimated using a one-sided significance level of 2.5% and a power of 80%. If an 18% dropout rate is included, then we need to recruit at least 110 subjects to this study to obtain a target sample size of 90 for evaluation.

The data analyses were performed using SAS 9.4 (SAS Institute, Inc., Cary, NC). All statistical tests were performed using a two-sided 0.05 level of significance. Data were analyzed on an intent-to-treat (ITT) basis. For the demographics, baseline assessments (SNAP-IV, CTRS-R: S, CGI-ADHD-S, and K-SASD-E ADHD symptoms), and adherence (average days of taking the medication per week), mean scores and SD were used for the continuous variables, while frequency and percentages were used for categorical variables. Descriptive statistics were used for the comparisons between the two treatment groups at baseline (Table 1). The baseline was defined as the last measurement obtained at or before randomization. The endpoint was defined as the last day of either Period 1 and Period 2. A paired *t*-test was used to examine the difference from baseline to the endpoints for Period 1 and Period 2, and this was done for each treatment group. Hierarchical linear mixed-effect models were carried out to address the lack of statistical independence of the repeated measurements obtained from the same participants during Period 1 and Period 2 using different treatments. We used mixed-effect models to test the treatment (ORADUR-MPH vs. placebo) differences at the two endpoints and the treatment differences in terms of the mean change from baseline (before randomization) to the endpoints of Period 1 and Period 2 (after taking the study drug for 2 weeks); these were used as efficacy measures. Patients with a baseline and at least one postbaseline measurement were included in the analysis. Cohen's *d* was used to calculate the effect sizes (the standardized difference between the two means) when comparing the efficacy measure scores between baseline and endpoints for each treatment group and at baseline and endpoints as well as the difference from the baseline to endpoint between the two treatment groups at each visit (Cohen 1988). The effect size of Cohen's *d* was considered small, medium, or large when the absolute value was 0.2–0.5, 0.5–0.8, or ≥0.8 (Lakens 2013), respectively. Fisher's exact test was used to compare the number of subjects with TEAEs between the two treatment groups. The frequency and percentage of TEAEs during study period II are presented for each treatment group.

**Table 1.** Summary Demographics and Baseline Attention-Deficit/Hyperactivity Disorder Symptoms of Two Groups (Intent-To-Treat Population)

Variable/mean (SD)	ORADUR^®^—placebo (N = 50)	Placebo—ORADUR^®^ (*N* = 50)	t/χ^2^	*p* Value
Age (years)	9.16 (2.42)	8.96 (2.39)	−0.416	0.678
Male, *n* (%)	37 (74.0%)	36 (72.0%)	0.051	0.822
Height (cm)	137.20 (14.05)	137.27 (15.64)	0.023	0.982
Weight (kg)	34.78 (13.61)	36.62 (13.69)	0.662	0.509
Dosage (mg/kg/day)	0.92 (0.22)	0.91 (0.33)	−0.128	0.899
SBP (mmHg)	103.98 (11.12)	104.98 (11.75)	0.426	0.671
DBP (mmHg)	65.81 (9.01)	61.62 (11.51)	−1.981	0.051
ADHD subtype, *n* (%)				
Combined type	37 (74.0%)	38 (76.0%)	0.053	0.817
Inattentive type	13 (26.0%)	12 (24.0%)		
ADHD symptoms (years)	0.06 (0.17)	0.03 (0.09)	−1.037	0.303
SNAP-IV—Teacher Form				
Inattention	15.4 (6.5)	15.4 (6.4)	0.000	1.000
Hyperactive/impulsivity	10.8 (8.7)	10.2 (7.5)	−0.345	0.731
Oppositional defiant	7.2 (7.7)	7.2 (6.8)	−0.055	0.956
Total score	33.4 (20.9)	32.8 (17.8)	−0.165	0.869
SNAP-IV—Parent Form				
Inattention	16.6 (4.8)	16.9 (4.6)	0.340	0.734
Hyperactive/impulsivity	12.5 (6.3)	12.5 (6.0)	0.065	0.948
Oppositional defiant	10.4 (5.3)	10.7 (6.0)	0.263	0.793
Total score	39.5 (12.8)	40.2 (13.5)	0.267	0.790
CTRS-R:S				
Inattention score	9.5 (6.4)	8.5 (5.6)	−0.770	0.443
Hyperactivity score	10.1 (4.8)	10.4 (4.9)	0.269	0.789
Oppositional score	8.7 (6.5)	8.5 (5.7)	−0.164	0.871
ADHD index score	11.9 (7.6)	12.2 (6.6)	0.211	0.833
CGI-ADHD-S	4.9 (1.1)	4.9 (1.0)	0.092	0.927
ADHD symptoms by K-SADS-E				
Inattention	8.6 (0.7)	8.4 (0.9)	−1.364	0.176
Hyperactivity-impulsiveness	6.4 (2.8)	6.3 (2.7)	−0.291	0.772
Father's education level				
Senior high or below	19 (38.0%)	21 (42.0%)	0.333	0.564
College or above	30 (60.0%)	28 (56.0%)		
Mother's education level				
Senior high or below	20 (40.0%)	16 (32.0%)	1.129	0.288
College or above	29 (58.0%)	31 (62.0%)		

ITT, intent to treat; ADHD, attention-deficit/hyperactivity disorder; SBP, systolic blood pressure; DBP, diastolic blood pressure; SNAP-IV, The Chinese version of the Swanson, Nolan, and Pelham, version IV; CTRS-R: S, The Chinese version of the Conners' Teacher Rating Scales Revised: Short; K-SADS-E, The Chinese version of the Kiddie Schedule for Affective Disorders and Schizophrenia for School-Age Children-Epidemiological; CGI-ADHD-S, The Clinical Global Impression-ADHD severity; SD, standard deviation.

## Results

### The characteristics of the participants and the administration of their medication

Of the 110 patients screened, 103 patients entered our study and were then randomized into the two treatment groups (Fig. 1). Of the seven patients who withdrew from the study, five did this due to an adverse effect of the medication. Of the 103 randomized patients, three were excluded and one hundred were included in the ITT population (Fig. 1). The 100 patients forming the ITT population were randomly assigned to the ORADUR-MPH (*n* = 50) and placebo (*n* = 50) groups for Period 1. There was no difference between the two treatment groups in terms of demographic data, vital signs, and the dosage of medication, or in all efficacy measures (Table 1). The lack of any difference between the two groups suggests that the randomization was successful.

Of 100 participants randomized, who completed Period 1, one from Period 1 ORADUR-MPH had consent withdrawal and one from Period 1 placebo group withdrew due to headache before Period 2. Hence, 99 participants received at least one dose of placebo during the crossover study period and these individuals were included in the placebo treatment group; furthermore, 99 participants received at least one dose of ORADUR-MPH during the crossover study period and these individuals were included in the ORADUR-MPH treatment group (Table 1, Fig. 1). The mean administered daily dose and daily dose per kilogram of ORADUR-MPH for the 99 participants were 30.56 mg/day (SD, 8.40 mg/day; range, 22–44 mg/day) and 0.92 mg/kg/day (SD, 0.28 mg/kg/day; range, 0.42–1.90 mg/kg/day), respectively. The mean administered daily dose and daily dose per kilogram of placebo for 99 participants were 30.64 mg/day (SD, 8.40 mg/day; range, 22–44 mg/day) and 0.92 mg/kg/day (SD, 0.28 mg/kg/day; range, 0.41–1.94 mg/kg/day), respectively. There were no significant group differences regarding the last mean daily dose (*p* = 0.942) and the mean daily dose per kilogram (*p* = 0.853).

### Primary efficacy measure (SNAP-IV-T)

**FIG. 2.**
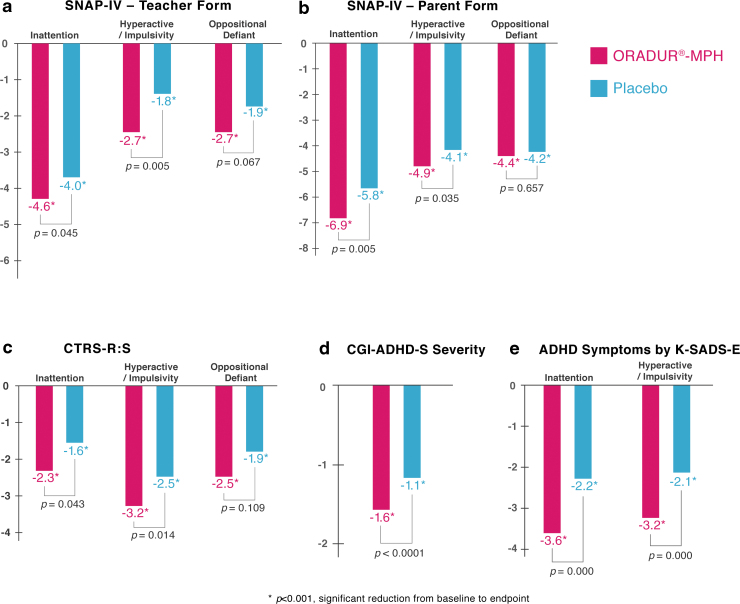
Reduction in the clinical symptoms from prerandomizaton to endpoint in the children with ADHD, who were randomly assigned to treatment with ORADUR-methylphenidate or with the placebo. **(a)** Teacher-rated SNAP-IV-Teacher Form; **(b)** parent-rated SNAP-IV-Parent Form; **(c)** teacher-rated CTRS-R: S; **(d)** investigator-rated CGI-ADHD-S; **(e)** investigator-rated ADHD symptoms. *p < 0.001 for mean symptom reduction from baseline to endpoint by paired *t-*test. The *p*-values are for comparisons of group differences in symptom reduction using a multilevel mixed model. ADHD, attention-deficit/hyperactivity disorder; CGI-ADHD-S, The Clinical Global Impression-ADHD severity; SNAP-IV, the Chinese version of the Swanson, Nolan, and Pelman-IV; CTRS-R: S, Conner's Teacher's Rating Scale-Revised: Short.

Both groups demonstrated significantly reduced symptom severity with respect to inattention, hyperactivity-impulsivity, ODD, and total SNAP-IV-T scores with small to medium effect sizes (Cohen's *d* values ranging from −0.41 to −0.74 for MPH and −0.26 to −0.61 for placebo, all *p*-values <0.001, see Table 2, Fig. 2). At the endpoint, the ORADUR-MPH group displayed less severe ADHD symptoms than the placebo group (*p* = 0.032–0.003), but with no difference in ODD symptoms (*p* = 0.081). The magnitude of symptom reduction from baseline to the endpoint was significantly greater for the ORADUR-MPH group than the placebo group with respect to inattention, hyperactivity-impulsivity, and total scores (*p* = 0.045–0.005), but not in ODD symptoms (*p* = 0.067).

**Table 2.** Symptom Change from Baseline (Pretitration) to Postbaseline (End Treatment) (Intent-To-Treat Population)

	ORADUR^®^-MPH (*N* = 99)					Placebo (*N* = 99)					Group differences			
	Baseline	Endpoint	Endpoint–baseline			Baseline	Endpoint	Endpoint–baseline			Endpoint		Endpoint–baseline	
Mean,* M *(SD)	*M* (SD)	*M* (SD)	*M* (SD)	Cohen's d	*p*	*M* (SD)	*M* (SD)	*M* (SD)	Cohen's d	*p*	Cohen's d	*p*	Cohen's d	*p*
SNAP-IV—Teacher Form														
Inattention	15.3 (6.4)	10.5 (6.6)	−4.6 (5.2)	−0.74	<0.0001	15.4 (6.5)	11.4 (6.6)	−4.0 (5.0)	−0.61	<0.0001	−0.14	0.032	−0.12	0.045
Hyperactive/impulsivity	10.4 (8.1)	7.3 (6.9)	−2.7 (5.1)	−0.41	<0.0001	10.5 (8.1)	8.5 (7.3)	−1.8 (4.9)	−0.26	0.0004	−0.17	0.003	−0.18	0.005
Oppositional defiant	7.1 (7.1)	4.3 (5.8)	−2.7 (5.1)	−0.43	<0.0001	7.2 (7.2)	5.1 (6.2)	−1.9 (4.5)	−0.31	0.0001	−0.13	0.081	−0.17	0.067
Total score	32.8 (19.2)	22.1 (17.3)	−10.0 (13.0)	−0.59	<0.0001	33.1 (19.4)	25.0 (17.9)	−7.7 (11.7)	−0.43	<0.0001	−0.16	0.005	−0.19	0.006
SNAP-IV–Parent Form														
Inattention	16.8 (4.7)	9.8 (4.1)	−6.9 (5.0)	−1.59	<0.0001	16.9 (4.6)	11.1 (5.2)	−5.8 (5.0)	−1.18	<0.0001	−0.28	0.004	−0.22	0.005
Hyperactive/impulsivity	12.5 (6.1)	7.6 (5.2)	−4.9 (4.7)	−0.86	<0.0001	12.5 (6.1)	8.3 (6.0)	−4.1 (4.1)	−0.69	<0.0001	−0.12	0.037	−0.18	0.035
Oppositional defiant	10.5 (5.7)	6.1 (4.5)	−4.4 (5.0)	−0.86	<0.0001	10.6 (5.7)	6.4 (5.0)	−4.2 (4.3)	−0.78	<0.0001	−0.06	0.612	−0.04	0.657
Total score	39.7 (13.1)	23.5 (11.5)	−16.2 (11.8)	−1.31	<0.0001	39.9 (13.1)	25.8 (14.0)	−14.1 (11.0)	−1.04	<0.0001	−0.18	0.021	−0.18	0.024
CTRS-R:S														
Inattention score	9.0 (6.0)	6.3 (5.5)	−2.3 (3.5)	−0.47	<0.0001	9.0 (6.0)	7.3 (6.0)	−1.6 (3.8)	−0.28	0.0001	−0.17	0.039	−0.19	0.043
Hyperactivity score	10.2 (4.8)	6.8 (4.7)	−3.2 (3.5)	−0.72	<0.0001	10.3 (4.8)	7.8 (5.0)	−2.5 (3.4)	−0.51	<0.0001	−0.21	0.015	−0.20	0.014
Oppositional score	8.6 (6.1)	5.7 (5.4)	−2.5 (4.1)	−0.50	<0.0001	8.6 (6.1)	6.6 (5.6)	−1.9 (4.1)	−0.34	<0.0001	−0.16	0.089	−0.15	0.109
ADHD index score	12.0 (7.1)	8.1 (6.5)	−3.6 (4.5)	−0.57	<0.0001	12.0 (7.1)	9.3 (6.8)	−2.6 (4.3)	−0.39	<0.0001	−0.18	0.010	−0.23	0.010
CGI-ADHD-S	4.9 (1.1)	3.3 (1.0)	−1.6 (1.2)	−1.52	<0.0001	4.9 (1.1)	3.9 (1.2)	−1.1 (1.2)	−0.87	<0.0001	−0.54	<0.0001	−0.42	<0.0001
ADHD symptoms by K-SADS-E														
Inattention	8.5 (0.8)	4.9 (3.0)	−3.6 (3.1)	−1.64	<0.0001	8.5 (0.8)	6.3 (2.8)	−2.2 (2.8)	−1.07	<0.0001	−0.48	0.000	−0.47	0.000
Hyperactivity/impulsiveness	6.3 (2.7)	3.1 (2.9)	−3.2 (3.3)	−1.14	<0.0001	6.4 (2.8)	4.3 (2.9)	−2.1 (2.8)	−0.74	<0.0001	−0.41	0.000	−0.36	0.000

ITT, intent to treat; ADHD, attention-deficit/hyperactivity disorder; SNAP-IV, The Chinese version of the Swanson, Nolan, and Pelham, version IV; CTRS-R: S, The Chinese version of the Conner's Teacher Rating Scales Revised: Short; CGI-ADHD-S, The Clinical Global Impression-ADHD severity; K-SADS-E, The Chinese version of the Kiddie Schedule for Affective Disorders and Schizophrenia for School-Age Children-Epidemiological; SD, standard deviation; MPH, methylphenidate.

### Secondary efficacy measures (SNAP-IV-P, CTRS-R: S)

Similar to the results for the SNAP-IV-T analysis, we found that both groups demonstrated significantly reduced inattention, hyperactivity-impulsivity, ODD, and total SNAP-IV-P scores with large to very large effect sizes for ORADUR-MPH (Cohen's *d* values −0.86 to −1.59) and medium to large effect sizes for placebo (Cohen's d values −0.70 to −1.18, see Table 2, Fig. 2). Significantly better efficacy was identified within the ORADUR-MPH group than within the placebo group when the group differences at endpoints and symptoms reduction from baseline to the endpoint for ADHD symptoms were compared (all *p*-values <0.05); however, this was not true for ODD symptoms.

When the teacher reports using the CTRS-R: S were examined, both groups showed significantly reduced scores for the Inattention, Oppositional, Hyperactivity, and the ADHD-index subscales with medium to large effect sizes for ORADUR-MPH and small effect sizes for the placebo (Table 2). Compared to the placebo group, ADHD children and adolescents treated with ORADUR-MPH showed a significant reduction in CTRS-R: S subscores at the endpoint and a greater reduction in subscores from baseline to the endpoint (all *p*-values <0.05), except for the Oppositional subscore (Table 2).

### Secondary efficacy measures (investigators' assessments)

Both groups showed significant decreases in the investigators' overall impression of ADHD symptom severity based on the CGI-ADHD-S, with much greater effect sizes for the ORADUR-MPH group (Cohen's *d* = −1.52) compared to the placebo group (Cohen's *d* = −0.87). Significant group differences in terms of overall ADHD symptoms were identified at the endpoints (*p* < 0.0001) and for the changes from baseline to the endpoint (*p* < 0.0001, see Table 2, Fig. 2).

When the results for the DSM-IV/DSM-5 ADHD symptom dimensions based on the K-SADS-E interviews by the investigators were examined, both groups demonstrated significant reductions in both inattention and hyperactivity-impulsivity symptoms, with large to very large effect sizes for the ORADUR-MPH group and medium to large effect sizes for the placebo group. The ORADUR-MPH group showed significantly fewer ADHD symptoms at the endpoints (both *p*-values <0.0001) as well as greater ADHD symptom reduction from the baseline to the endpoint (both *p*-values <0.0001), both compared to the placebo group (Table 2, Fig. 2).

### Group comparisons from baseline to precrossover endpoint

We also conducted the ITT analysis using the end of the first treatment before crossover as an endpoint (Supplementary Table S1). Both groups (*n* = 50 for each group) showed significantly reduced symptom severity in all the efficacy measures with small to large effect sizes and a consistent trend of larger effect sizes for each efficacy measure in the ORADUR-MPH group (Cohen's *d* values ranging from −0.38 to −1.65, all *p*-values ≤0.001) than the placebo group (Cohen's *d* values ranging from −0.23 to −1.09, *p*-values ranging from 0.018 to <0.001), regardless of statistical significance levels of group comparison. At the endpoint, the ORADUR-MPH group displayed less severe ADHD symptoms than the placebo group with small effect sizes (absolute Cohen's *d* > 0.20) in inattention, hyperactivity-impulsivity, and total scores of SNAP-IV-P form, and investigator's interview of ADHD symptoms. However, none of these comparisons reached statistical significance. Similarly, there was a trend that the magnitudes of symptom reduction from baseline to the endpoint were greater for the ORADUR-MPH group than the placebo group with small effect sizes (absolute Cohen's *d* > 0.20) in hyperactivity-impulsivity, and total scores of SNAP-IV-T and SNAP-IV-P, inattention score of the CTRS-R: S, and investigator's interview of ADHD symptoms (Supplementary Table S1). However, only the group comparison of DSM-5 inattention symptoms reached a statistically significant difference (*p* < 0.05). The small sample size may partially explain the low power to detect group differences.

### Safety measures

When the incidence of TEAEs and AEs observed in the study were examined, 278 TEAEs were reported involving 79 (71.8%) participants for the ORADUR-MPH group and 10 (9.9%) participants for the placebo group. All of the TEAEs were determined to be either Grade 1 or Grade 2 in terms of severity by the investigators. Furthermore, among the TEAEs determined to be drug-related AE by investigators, 211 TEAEs were observed in 68 (61.8%) participants while they were receiving ORADUR-MPH, while seven TEAEs were observed in four (4.0%) participants while receiving placebo.

A summary of the incidence of TEAEs is presented in Table 3. The most common TEAE (ORADUR-MPH vs. placebo, %) was a decrease in appetite (48.2% and 1.0%), followed by insomnia (20.0% and 0.0%), nausea (13.6% and 0.0%), headache (8.2% and 1.0%), dizziness (6.4% and 0.0%), upper abdominal pain (4.5% and 1.0%), and palpitation/tachycardia (4.5% and 1.0%). Significant group differences in TEAEs were only found in decreased appetite, insomnia, nausea, headache, and dizziness (all *p*-values <0.05, see Table 3). No drug-related severe AE was reported.

**Table 3.** Summary of Incidence of Drug-Related Adverse Events (Safety Population)

Adverse events	ORADUR^®^-MPH**(*N* = 110)		Placebo**(*N* = 101)		Fisher's exact
	Event	Participant (%)	Event	Participant (%)	*p*-Value
Decreased appetite	81	53 (48.2)	1	1 (1.0)	**<0.0001**
Gastrointestinal symptoms	38	22 (20.0)	3	1 (1.0)	**<0.0001**
Nausea	20	15 (13.6)	0	0 (0.0)	**<0.0001**
Vomiting	4	4 (3.6)	0	0 (0.0)	0.1228
Stomachache	7	5 (4.5)	1	1 (1.0)	0.2146
Abdominal discomfort	3	2 (1.8)	0	0 (0.0)	0.4985
Abdominal pain	2	2 (1.8)	0	0 (0.0)	0.4985
Diarrhea	2	2 (1.8)	2	1 (1.0)	1.0000
Neurological symptoms					
Dizziness	13	7 (6.4)	0	0 (0.0)	**0.0147**
Headache	12	9 (8.2)	1	1 (1.0)	**0.0197**
Palpitations/tachycardia	5	5 (4.5)	0	0 (0.0)	0.0607
Psychiatric conditions					
Sleep problems	34	22 (20.0)	0	0 (0.0)	**<0.0001**
Agitation	4	4 (3.6)	0	0 (0.0)	0.1228
Moody	2	2 (1.8)	0	0 (0.0)	0.4985
Irritability	3	3 (2.7)	0	0 (0.0)	0.2478
Tics	2	2 (1.8)	0	0 (0.0)	0.4985
Decreased weight	4	4 (3.6)	0	0 0.00	0.1228
Other general symptoms					
Chest discomfort/pain	3	3 (2.7)	0	0 (0.0)	0.2478
Dyspnea	1	1 (0.9)	0	0 (0.0)	1.0000
Fatigue	2	2 (1.8)	0	0 (0.0)	0.4985
Edema peripheral	1	1 (0.9)	0	0 (0.0)	1.0000
Thirst	1	1 (0.9)	0	0 (0.0)	1.0000
Musculoskeletal stiffness	1	1 (0.9)	0	0 (0.00)	1.0000
Asthenia	1	1 (0.9)	0	0 (0.0)	1.0000
Oropharyngeal pain	1	1 (0.9)	0	0 (0.0)	1.0000

Bold values: *p* < 0.05.

MPH, methylphenidate.

No significant change in any of the other safety assessments, such as vital signs and laboratory safety profiles, was found, except for changes in mean weight of the participants. At the endpoint, the MPH group had gained weight (mean ± SD, 0.28 ± 1.37 kg; *p* = 0.010), as had the placebo group (mean ± SD, 0.31 ± 1.40 kg; *p* = 0.007); both changes were statistically significant. Neither of the treatment groups was found to show significant changes in their vital signs, weight, height, or laboratory safety profiles.

## Discussion

As the first report of the clinical efficacy and tolerability of ORADUR-MPH, our placebo-controlled crossover trial found that ORADUR-MPH significantly reduced symptoms of inattention, hyperactivity, impulsivity, ADHD, and ODD with 2-week treatment, regardless of informants. ORADUR-MPH would be efficacious in that there is a significantly greater magnitude of improvement in symptoms compared to the placebo when inattention, hyperactivity, and impulsivity are assessed regardless of the source of the information. However, there was no group difference when improvements in ODD symptoms were examined based on the parent's and the teacher's ratings. Our findings of a significant improvement in ADHD core symptoms and overall clinical symptoms when individuals are treated with ORADUR-MPH compared to treatment with placebo are consistent with previous extensive research on a range of different formulations of MPH that have been carried out all over the world (Chan et al. 2016; Kim et al. 2017; Pliszka et al. 2017; Wigal et al. 2017; Lam et al. 2019), including Taiwan (Gau et al. 2006b, 2008; Chou et al. 2009; Shang et al. 2015; Ni et al. 2017). Two medications (MPH and atomoxetine) have been approved for the treatment of ADHD in a wide range of countries; these treatments are based on the hypothesis that dopaminergic and noradrenergic dysregulation are involved in ADHD (Del Campo et al. 2011). Of these, MPH has been used to treat ADHD since the 1950s. MPH binds to and inhibits the DAT with a high affinity (Schenk 2002) and the norepinephrine transporter with a low affinity (Markowitz et al. 2006); this then alters dopamine and norepinephrine transport across the synaptic membrane (Kasparbauer et al. 2015). Numerous studies, including this study, have provided strong evidence to support the fact that MPH is able to reduce the three core symptoms of ADHD, namely inattention, hyperactivity, and impulsivity (Gau et al. 2006a; Shang et al. 2015; Childress et al. 2017; Clavenna and Bonati 2017). Such symptom improvement seems to be mediated by MPH bringing about an improvement in sustained attention (Tucha et al. 2006; Bedard et al. 2015; Chou et al. 2015), response inhibition (Broyd et al. 2005; Kratz et al. 2009; Pauls et al. 2012; Dougherty et al. 2016; Pievsky and McGrath 2018), cognitive impulsivity, and motor impulsivity (Kratz et al. 2009; Chou et al. 2015; Dougherty et al. 2016).

Evidence of the effectiveness of MPH in terms of the IR formulation has been available for several decades. However, because of the short half-life of MPH (2–3 hours), the IR formulation usually needs to be administered twice or thrice per day to maintain therapeutic efficacy (Pelham et al. 2001). Due to the need for multiple doses, disease stigma, poor drug adherence, self-medication, and the diversion caused by IR-MPH are common (Sanchez et al. 2005; Gau et al. 2006b; Atzori et al. 2009), and there has been much research targeted at the development of an extended-release formulation that will have improved safety, better tolerability, and greater convenience (Childress 2017). Despite substantial evidence indicating improved clinical symptoms, the different duration of the drug's effects (OROS MPH, 10–12 hours; Ritalin LA, 6–8 hours) and variation in the drugs' profiles (e.g., OROS MPH has an ascending profile) seem to have created a need for an extended-release formulation that has both the benefits described above and is able to fulfill the unmet needs of patients with ADHD (Lyseng-Williamson and Keating 2002; Coghill et al. 2013; Maldonado 2013; Childress et al. 2017; Clavenna and Bonati 2017; Pliszka et al. 2017).

ORADUR-MPH contains MPH hydrochloride as the active pharmaceutical ingredient, and this has been incorporated into the ORADUR drug delivery platform, which utilizes a high-viscosity base component, namely sucrose acetate isobutyrate, along with other excipients; these are able to bring about the controlled release of MPH (Cortese et al. 2017). ORADUR-MPH was designed to be a once-a-day, abuse-resistant, and tamper-resistant product that is able to bring about a steady delivery of MPH throughout the day; this will help to minimize the well-known risks associated with the peaks and valleys of IR-MPH treatment, while providing the same or improved therapeutic benefits compared to other commercially available forms of MPH (Cortese et al. 2017). This study is the first to prove the short-term efficacy and safety of ORADUR-MPH in children and adolescents with ADHD.

In addition to affecting ADHD's core symptoms, some studies have also demonstrated how MPH is able to effectively bring about improvements in a wide range of emotional and behavioral problems (Shih et al. 2019), as well as allowing better overall functioning at school (Shang et al. 2019). Surprisingly, although MPH in this study has been demonstrated to bring about improvements in ODD symptoms as reported by the parents and teachers in the study, the effects of MPH on ODD symptoms were not significantly different to those of the placebo. Several clinical trials (Garg et al. 2015; Masi et al. 2017; Golubchik et al. 2018; Pan et al. 2018; Sultan et al. 2019), including ours (Shang et al. 2015; Shih et al. 2019), have reported that MPH is able to reduce ODD symptoms. Nevertheless, there is a significant amount of evidence available, supporting the idea that ADHD patients with ODD symptoms or an ODD diagnosis seem to benefit from the addition of atypical antipsychotics, such as risperidone (Masi et al. 2017; Sultan et al. 2019), quetiapine (Sultan et al. 2019), and aripiprazole (Pan et al. 2018; Sultan et al. 2019), to their MPH treatment (Masi et al. 2017; Golubchik et al. 2018); these additional useful interventions also include psychosocial interventions (Abikoff et al. 2004; Van der Oord et al. 2008; Chan et al. 2016; Suzer Gamli and Tahiroglu 2018). Consistent with previous studies, MPH may help to reduce the symptom severity of ODD, but combining this drug with intensive psychosocial intervention and, if needed, atypical antipsychotic treatment, is likely to bring about greater benefits to ADHD patients with ODD symptoms.

The AEs observed in previous pharmacokinetic studies of methylphenidate have included palpitation, headache, nausea, dizziness, diarrhea, fever, hypotension, urinary tract infection, and an increase in blood pressure. All of these AEs are mild and tolerable (Swanson et al. 2004; Muniz et al. 2008; Wigal et al. 2017). The most common TEAE observed in the MPH group was a decreased appetite (48.2%). The other common TEAEs (>5.0%) observed among the participants treated with ORADUR-MPH are sleep problems (20.0%), nausea (13.6%), headache (8.2%), and dizziness (6.4%). No statistically significant body weight loss was observed among participants who received ORADUR-MPH by the end of study. Still, instead, they showed significant body weight gains, suggesting that the complaints of decreased appetite may not be directly related to the ORADUR-MPH treatment or that the decreased appetite did not have an influence on the weight gain detected among the participants. Another explanation for the report of decreased appetite in almost half of the patients treated with MPH may be that the participants and their parents were familiar with a decreased appetite as the most commonly reported adverse effect of MPH treatment (Storebo et al. 2015, 2018; Childress et al. 2017; Clavenna and Bonati 2017; Holmskov et al. 2017). The need for parents to report AEs may affect the incidence of AEs in the clinical studies that are targeting children and adolescents (Holmskov et al. 2017). A well-known adverse effect may increase the likelihood of report bias. Nevertheless, throughout the study, no serious AE was reported.

Despite various different formulations (Childress et al. 2017; Clavenna and Bonati 2017), the reports of MPH adverse effects generally include decreased appetite, nausea, stomach ache (upper abdominal pain), sleep problems, headache, dizziness, abdominal pain, vomiting, and tics. Therefore, most of the common TEAEs of ORADUR-MPH have been commonly observed in other similar marketed products involving extended-release of MPH (Swanson et al. 2004; Muniz et al. 2008; Wigal et al. 2017). When compared to the TEAEs identified in our previous head to head (OROS-MPH and IR-MPH) randomized clinical trial (Gau et al. 2006b), similar rates of decreased appetite after treatment for 2 weeks were reported, but there was a significant reduction in other adverse effects such as stomach aches, headaches, dizziness, tics, an anxious mood, and nail biting. Hence, ORADUR-MPH would seem to have fewer AEs than OROS or other IR forms of MPH.

The other safety measures, including laboratory tests and vital signs, also provide evidence to support the safety and tolerability of ORADUR-MPH. Surprisingly, a decline in body weight, which has been commonly observed in other similar marketed MPH drug formulations, was not observed with ORADUR-MPH. Our study did not support the idea that a decrease in body weight is associated with ORADUR-MPH treatment. In contrast, we found both the MPH group and placebo groups showed a slight increase in body weight after a 2-week treatment and that this change was statistically significant. Overall, ORADUR-MPH is safe and tolerable for the treatment of children and adolescents with ADHD.

### Strengths and limitations

Several features of this clinical trial make up its strengths. These include the study design (a randomized, double-blind, placebo-controlled two-way crossover trial) that uses paired analysis due to the lack of independence across the same participants (Li et al. 2015), it being the first study to investigate a new extended-release formulation of MPH (ORADUR-MPH), the use of multiple informants (parent, teacher, and investigator), and the combining of interviews with internationally well-known standard instruments involving questionnaires. It is worth noting that the teacher reports using SNAP-IV form the primary efficacy measure and that this measure demonstrated the significant superiority of ORADUR-MPH over placebo. Finally, this trial has a very low dropout rate, and there was also little missing data, both of which are significant methodological strengths.

This study, however, does have some limitations. These include the short treatment period, the lack of control regarding the time of day that reports are completed, the exclusion of ADHD patients with comorbidities such as other psychiatric disorders, the fact that the recruited population consisted largely of male subjects, and the lack of either a parallel placebo-controlled trial or a head-to-head comparison with standard psychopharmacotherapic treatment for ADHD.

First, our findings provide evidence of acute efficacy, but do not investigate the value of longer-term therapy. The brief treatment period in this study hampered our ability to make inferences regarding long-term efficacy and safety of ORADUR-MPH for treating children and adolescents with ADHD in the study population in Taiwan. Second, although the participants were assessed by the investigators and reported by the parents within a window of days of the various assessments and the teachers were asked to complete the Chinese SNAP-IV exactly the day before specific visits, there is no complete information on the exact days that these various ratings were completed. Third, this study excluded patients with some other psychiatric disorders, including depression and anxiety, which, to some extent, are often comorbid with ADHD. The above, combined with the predominantly male sample, and the fact that the sample was restricted to patients at only three medical centers in Taiwan, limit the ability to generalize our findings to a wide clinical population suffering from ADHD. Fourth, we used a two-way crossover study design without a washout period rather than a parallel study design (Krogh et al. 2019). We took advantage of the fewer samples that such a two-way crossover study needs, as well as the ability to obtain more precise estimates of treatment effects by removing any biological and methodological variation that are present within the crossover study design (Mills et al. 2009; Li et al. 2015; Krogh et al. 2019). Importantly, MPH has a short half-life, short-lived effectiveness, and a very limited carryover effect, and in this context, a meta-analysis has reported that four fifths of MPH clinical trials have used a crossover design (Krogh et al. 2019). Like many previous crossover studies, we did not include a washout period; such a period has been included in only 27 out of 147 crossover studies that have been reviewed. These washout periods have ranged from 1 to 14 days (Krogh et al. 2019). Our randomization procedure should have also diminished any period effect (Richens et al. 2001; Li et al. 2015). Fifth, although the efficacy of ORADUR-MPH has been demonstrated in this study and the tolerability was good, this study does not provide information about a head-to-head comparison of ORADUR-MPH with other formulations of MPH (Gau et al. 2006b; Coghill et al. 2013) or with atomoxetine (Garg et al. 2015; Ni et al. 2016, 2017; Shang et al. 2016), both of which are currently used for treating ADHD. Finally, like all the MPH treatment studies, methylphenidate gives rise to several easily recognizable AEs, one such being decreased appetite; such well-known adverse effects may lead to a loss of blinding and thus influence rating of symptom, particularly when this is carried out by parents and assessors who have knowledge of reported adverse effects.

## Conclusions

Our findings indicate that ORADUR-MPH is an efficacious, safe, and well-tolerated medication for treating children and adolescents with ADHD and that it does this by reducing their ADHD core symptoms without serious AEs. It provides another treatment option for patients with ADHD. It will be very useful in the future if the efficacy of ORADUR-MPH on other emotional and behavioral problems (Shih et al. 2019), social functionality (Shang et al. 2019), neuropsychological performance (Ni et al. 2013), and brain functioning (Chou et al. 2015) is investigated. It would also be very helpful to conduct head-to-head studies to obtain data on the comparative efficacy of the various MPH formulations available (Coghill et al. 2013) and to carry out a comparative study against atomoxetine (Garg et al. 2015; Ni et al. 2016, 2017; Shang et al. 2016).

## Clinical Significance

This is the first clinical trial to investigate the safety, tolerability, and efficacy of ORADUR-MPH and our study provides strong evidence to support ORADUR-MPH as an efficacious treatment that reduces inattention, hyperactivity, and impulsivity of ADHD patients. The treatment thus improves the overall clinical symptoms associated with ADHD compared to the placebo, and this is regardless of the type of informant, namely parent, teacher, or investigator. Hence, ORADUR-MPH provides another choice when the use of an extended release formulation of MPH is indicated; this should help to meet the unmet needs of children and adolescents with ADHD. However, whether ORADUR-MPH is more effective than other formulations of MPH needs head-to-head studies to provide data on the comparative efficacy of the various extended-release formulations of methylphenidate that are available.

## Supplementary Material

Supplemental data
