# Supplementary material for: A Randomized, Double-Blind, Placebo-Controlled, Two-Way Crossover Clinical Trial of ORADUR-Methylphenidate for Treating Children and Adolescents with Attention-Deficit/Hyperactivity Disorder
Source: J Child Adolesc Psychopharmacol. 2021 Apr 16;31(3):164–78. doi: 10.1089/cap.2020.0104 (PMC8066345; doi:10.1089/cap.2020.0104)
Supplement: Supplemental data [file Supp_TableS1.docx]

***Supplementary Table. Symptom Change from Baseline (Pre-titration) to Study V1 (Pre-crossover)***

|  | **ORADUR^®^-MPH**  **(N=50)** | | | | | | | | | **Placebo**  **(N=50)** | | | | | **Group Differences** | | | | | | | |
| --- | --- | --- | --- | --- | --- | --- | --- | --- | --- | --- | --- | --- | --- | --- | --- | --- | --- | --- | --- | --- | --- | --- |
|  | **Baseline** | | **Endpoint** | | **Endpoint-Baseline** | | | | | **Baseline** | **Endpoint** | **Endpoint-Baseline** | | |  | **Endpoint** | | | **Endpoint-Baseline** | | | |
| **Mean, M (SD)** | **M (SD)** | | **M (SD)** | | **M (SD)** | | ***Cohen's d*** | | **p** | **M (SD)** | **M (SD)** | **M (SD)** | ***Cohen's d*** | **p** |  | ***Cohen's d*** | | **p** | | ***Cohen's d*** | | **p** |
| **SNAP-IV – Teacher Form** | |  | |  | |  | |  | |  |  |  |  |  |  | |  |  | | |  |  |
| Inattention | 15.4 (6.5) | | 10.7 (6.8) | | -4.6 (4.8) | | -0.71 | | <.0001 | 15.4 (6.4) | 11.3 (6.0) | -4.1 (5.3) | -0.66 | <.0001 |  | -0.09 | | 0.657 | | -0.10 | | 0.628 |
| Hyperactive/Impulsivity | 10.8 (8.7) | | 7.7 (7.4) | | -3.0 (5.7) | | -0.38 | | 0.001 | 10.2 (7.5) | 8.5 (7.1) | -1.6 (4.6) | -0.23 | 0.018 |  | -0.11 | | 0.603 | | -0.27 | | 0.202 |
| Oppositional Defiant | 7.2 (7.7) | | 4.5 (6.6) | | -2.7 (5.2) | | -0.38 | | 0.001 | 7.2 (6.8) | 5.0 (5.9) | -1.9 (4.2) | -0.35 | 0.003 |  | -0.08 | | 0.708 | | -0.17 | | 0.401 |
| Total Score | 33.4 (20.9) | | 22.9 (19.1) | | -10.3 (13.7) | | -0.52 | | <.0001 | 32.8 (17.8) | 24.8 (17.0) | -7.7 (10.6) | -0.46 | <.0001 |  | -0.11 | | 0.620 | | -0.21 | | 0.288 |
| **SNAP-IV – Parent Form** |  | |  | |  | |  | |  |  |  |  |  |  |  |  | |  | |  | |  |
| Inattention | 16.6 (4.8) | | 10.0 (4.0) | | -6.6 (5.0) | | -1.49 | | <.0001 | 16.9 (4.6) | 11.3 (5.9) | -5.6 (5.4) | -1.06 | <.0001 |  | -0.26 | | 0.208 | | -0.19 | | 0.358 |
| Hyperactive/ Impulsivity | 12.5 (6.3) | | 7.5 (5.3) | | -4.9 (4.9) | | -0.86 | | <.0001 | 12.5 (6.0) | 8.7 (6.3) | -3.8 (4.1) | -0.62 | <.0001 |  | -0.21 | | 0.320 | | -0.24 | | 0.235 |
| Oppositional Defiant | 10.4 (5.3) | | 6.0 (3.7) | | -4.3 (5.1) | | -0.96 | | <.0001 | 10.7 (6.0) | 6.8 (5.4) | -3.9 (4.6) | -0.68 | <.0001 |  | -0.17 | | 0.438 | | -0.08 | | 0.667 |
| Total Score | 39.5 (12.8) | | 23.6 (10.5) | | -15.8 (12.4) | | -1.36 | | <.0001 | 40.2 (13.5) | 26.8 (15.5) | -13.4 (11.5) | -0.92 | <.0001 |  | -0.24 | | 0.235 | | -0.20 | | 0.305 |
| **CTRS-R:S** |  | |  | |  | |  | |  |  |  |  |  |  |  |  | |  | |  | |  |
| Inattention score | 8.7 (6.5) | | 5.8 (5.9) | | -2.8 (4.3) | | -0.47 | | <.0001 | 8.5 (5.7) | 6.4 (5.2) | -2.0 (3.6) | -0.38 | 0.000 |  | -0.11 | | 0.594 | | -0.20 | | 0.322 |
| Hyperactivity score | 10.1 (4.8) | | 7.0 (5.0) | | -3.1 (3.4) | | -0.63 | | <.0001 | 10.4 (4.9) | 7.7 (5.0) | -2.7 (3.3) | -0.55 | <.0001 |  | -0.14 | | 0.524 | | -0.12 | | 0.484 |
| Oppositional score | 9.5 (6.4) | | 6.9 (6.1) | | -2.5 (4.2) | | -0.42 | | <.0001 | 8.5 (5.6) | 6.0 (4.5) | -2.5 (4.2) | -0.49 | <.0001 |  | 0.17 | | 0.382 | | 0.00 | | 0.981 |
| ADHD index score | 11.9 (7.6) | | 8.3 (7.1) | | -3.5 (4.6) | | -0.49 | | <.0001 | 12.2 (6.6) | 9.0 (6.2) | -3.0 (4.0) | -0.50 | <.0001 |  | -0.11 | | 0.583 | | -0.12 | | 0.552 |
| **CGI-ADHD-S** | 4.9 (1.1) | | 3.5 (1.1) | | -1.4 (1.1) | | -1.27 | | <.0001 | 4.9 (1.0) | 3.7 (1.2) | -1.2 (1.4) | -1.09 | <.0001 |  | -0.17 | | 0.386 | | -0.16 | | 0.471 |
| **ADHD Symptoms by K-SADS-E** | | | |  | |  | |  | |  |  |  |  |  |  | |  |  | | |  |  |
| Inattention | 8.6 (0.7) | | 5.0 (3.0) | | -3.6 (3.0) | | -1.65 | | <.0001 | 8.4 (0.9) | 6.0 (3.1) | -2.4 (3.1) | -1.05 | <.0001 |  | -0.33 | | 0.108 | | -0.39 | | 0.050 |
| Hyperactivity/Impulsiveness | 6.4 (2.8) | | 3.1 (3.1) | | -3.4 (3.3) | | -1.12 | | <.0001 | 6.3 (2.7) | 3.9 (2.9) | -2.4 (2.8) | -0.86 | <.0001 |  | -0.27 | | 0.183 | | -0.33 | | 0.123 |

Abbreviations: ITT population: Intent-to-treat population; SNAP-IV: The Chinese version of the Swanson, Nolan, and Pelham, version IV scale; CTRS-R:S: The Chinese version of the Conner’s Teacher Rating Scales Revised; CGI-ADHD-S: The Clinical Global Impression-ADHD severity; K-SADS-E: The Chinese version of the Kiddie Schedule for Affective Disorders and Schizophrenia for School-Age Children–Epidemiological Version.
